# JAK2 in Myeloproliferative Neoplasms: Still a Protagonist

**DOI:** 10.3390/ph15020160

**Published:** 2022-01-28

**Authors:** Michael Stephan Bader, Sara Christina Meyer

**Affiliations:** 1 Division of Hematology, University Hospital Basel, CH-4031 Basel, Switzerland; michaelstephan.bader@usb.ch; 2 Department of Biomedicine, University Hospital Basel and University of Basel, CH-4031 Basel, Switzerland

**Keywords:** JAK2, myeloproliferative neoplasms, JAK2 inhibitors, resistance

## Abstract

The discovery of the activating V617F mutation in Janus kinase 2 (JAK2) has been decisive for the understanding of myeloproliferative neoplasms (MPN). Activated JAK2 signaling by *JAK2*, *CALR,* and *MPL* mutations has become a focus for the development of targeted therapies for patients with MPN. JAK2 inhibitors now represent a standard of clinical care for certain forms of MPN and offer important benefits for MPN patients. However, several key aspects remain unsolved regarding the targeted therapy of MPN with JAK2 inhibitors, such as reducing the MPN clone and how to avoid or overcome a loss of response. Here, we summarize the current knowledge on the structure and signaling of JAK2 as central elements of MPN pathogenesis and feature benefits and limitations of therapeutic JAK2 targeting in MPN.

## 1. Introduction

Janus kinases (JAKs) constitute a family of intracellular, non-receptor tyrosine kinases. They are associated with their corresponding cell surface receptors and transduce signals from cytokines, as well as some hormones, such as growth hormone and prolactin [1]. Mediation of extracellular signals via JAKs activates intracellular messenger pathways, impacting hematopoiesis, metabolism, and immune responses [2,3,4]. The discovery that most *BCR-ABL1*-negative myeloproliferative neoplasms (MPN) carry an activating *JAK2* mutation has moved this tyrosine kinase into the center of interest and has led to refined diagnostic criteria for these disease entities, development of new prognostic models, and the introduction of JAK inhibitors into clinical practice. Here, we review the role of JAK2 in myeloproliferative neoplasms (Supplementary Materials).

## 2. JAK2 Structure and Signaling

**Figure 1:**
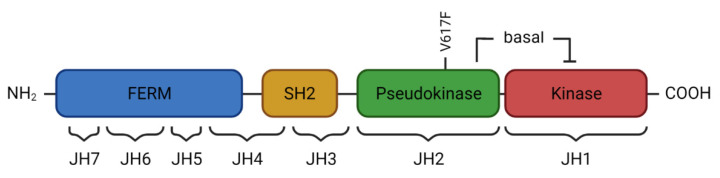
**Schematic representation of the JAK2 domain structure.** Seven Janus homology domains (JH1–JH7) constitute the *N*-terminal FERM and SH2 domain associating with the intracellular domains of cell surface receptors as well as the C-terminal pseudokinase and kinase domain of JAK2. The JAK2 V617F mutation locates to the pseudokinase domain, interfering with the basal inhibitory effect of the pseudokinase on the kinase domain, leading to constitutive JAK2 activation.

JAK2 and the additional Janus kinase family members JAK1, JAK3, and TYK2 share a similar structure with seven Janus homology (JH)-domains (JH1–JH7, Figure 1) [5,6]. The *N*-terminal FERM-domain (JH5–JH7), named after the proteins Band 4.1, ezrin, radixin, and moesin, and the Src homology 2 (SH2) domain mediate binding to the cytoplasmic portion of the associating cytokine receptors [7]. The C-terminal part of JAK2 contains the JH1 domain, which represents the catalytically active tyrosine kinase domain, and the so-called pseudokinase domain (JH2). The close proximity of the two kinase domains has led to the JAKs being named after the two-faced god Janus. Characterization of the structure, function, and regulation of JAK2 has been complicated by the challenging purification of the protein and its subunits. In case of the JH2-domain, only the exchange of three hydrophobic amino acids enabled the formation of protein crystals, which could subsequently be analyzed [8]. It has become clear that the pseudokinase domain has a regulatory impact on the JAK2 kinase domain via phosphorylation of S523 and Y570, mediating basal autoinhibition, which is reversed by stimulation of the associated cytokine receptor [9,10,11].

**Figure 2:**
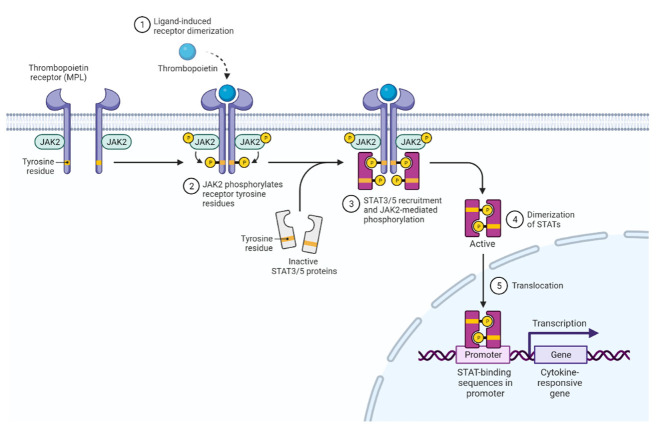
**The** **JAK-STAT** **signaling** **pathway** **in** **MPN.**

JAK2 activation occurs upon ligand binding to hematopoietic cytokine receptors such as the erythropoietin receptor (EPOR), thrombopoietin receptor (TPOR or MPL), or the granulocyte-macrophage colony-stimulating factor (GM-CSFR), as well as IL-3 and IL-5 receptors. JAK2 is also the mediator of signaling from growth hormone, leptin, and prolactin receptors [1] (Figure 2). Receptor dimerization enables transphosphorylation of the tandem tyrosines Y1007 and Y1008 in the activation loop of the JAK2 kinase domain, increasing catalytic activity [12].

Activated JAK2 mediates phosphorylation of its cognate receptors at the cytoplasmic tail and the generated phosphotyrosines serve as docking sites for SH2 domain-containing signaling proteins such as the signal transducer and activator of transcription (STATs). In MPN, STAT3 and STAT5 are phosphorylated, dimerize and translocate to the nucleus to regulate transcriptional programs, promoting cell proliferation, differentiation, and survival [3,13].

**Figure 3:**
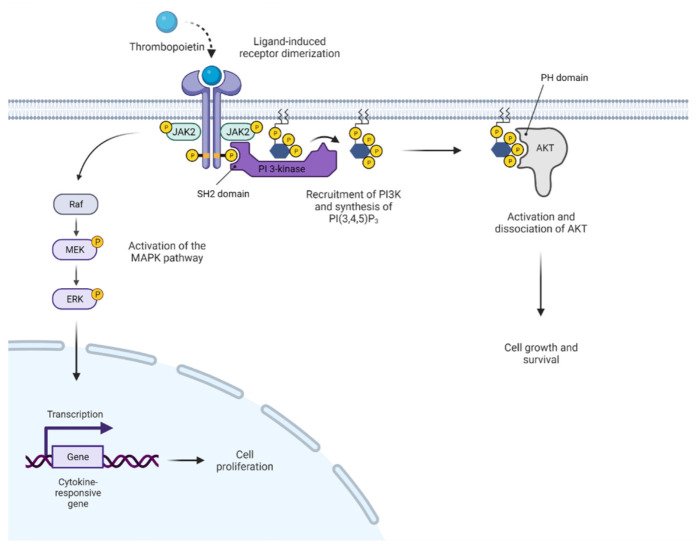
**JAK2** **signaling** **also** **activates** **MAPK** **and** **PI3K/Akt** **pathways,** **promoting** **cell** **proliferation** **and** **survival.**

In addition, JAK2 also activates the mitogen-activated protein kinase (MAPK) pathway, which is implicated in many cancers (Figure 3, reviewed in Ref. [14]) and contains three evolutionarily conserved protein kinases including RAF, MEK1/2, and ERK1/2, as well as the PI3K/AKT/mTOR pathway (reviewed in Ref. [15]), which further enhance cell proliferation and survival. Phosphorylation of PIM1 kinase and the Bcl-2 family member protein Bad inhibit apoptotic cell death and promote cell survival [2].

JAK2 signaling activity is negatively regulated by several factors, such as via negative feedback through suppressors of cytokine signaling (SOCS) proteins [16]. While the eight SOCS family members SOCS1-7 and the cytokine-inducible SH2-containing protein (CIS) are recruiting ubiquitin ligases (E3) to target JAK2 and other proteins for degradation, it has been shown that SOCS3 is able to directly inhibit JAK2 kinase activity via complex formation. In addition, phosphatases including SHP1 and SHP2 as well as other protein tyrosine phosphatases (PTPs) contribute to JAK2 inactivation via dephosphorylation of JAK2 and the associated receptors [17]. JAK2 signaling also induces LNK (also known as SH2B3) and other members of the SH2B family, which are established regulators of JAK2. LNK has been shown to bind to phosphorylated JAK2 via its SH2 domain in an erythropoietin- or thrombopoietin-dependent manner, thereby inhibiting erythropoiesis and thrombopoiesis [18]. Negative regulators of STATs, such as the protein inhibitors of STATs (PIAS), further contribute to limiting the activation of JAK2-STAT signaling via ubiquitinylation and subsequent proteasomal degradation [2].

## 3. Activated JAK2 Signaling in MPN

The *JAK2* V617F gain-of-function mutation was identified in 2005 by several laboratories [19,20,21,22], and it has greatly advanced our insight into the pathogenesis of *BCR-ABL1*-negative MPN. It has also facilitated MPN diagnosis and has highlighted the key role of activated JAK2 signaling, thus providing a basis for rationally designed targeted therapies. The *JAK2* V617F mutation is located in exon 14, i.e., in the pseudokinase domain of JAK2 on chromosome 9p24, and consists of a single nucleotide substitution (G1849T). Crystal structure analyses of the V617F mutant JAK2 pseudokinase domain have shown that steric alterations interfering with the inhibitory effect of the pseudokinase on the kinase domain induce constitute activation of JAK2 [8,23]. Consequently, dysregulated STAT, as well as PI3K/AKT and MAPK pathway signaling, occurs downstream of the EPOR, MPL (or TPOR), and GM-CSFR, resulting in excessive production of mature myeloid blood cells.

The *JAK2* V617F mutation is found in the majority of MPN patients including all three subtypes, such as in 95% of patients with polycythemia vera (PV) and 50–60% of patients with essential thrombocythemia (ET) or myelofibrosis (MF) (Table 1) [24]. In addition, several missense mutations, small insertions, or deletions in exon 12 of *JAK2* were identified in 2007. *JAK2* exon 12 mutations are encountered in 2–3% of patients with PV and are absent in ET and PMF [25]. Patients may show isolated and more pronounced erythrocytosis independent of the specific exon 12 mutation. In contrast, patients with the *JAK2* V617F mutation often present with trilineage hyperplasia. Nevertheless, disease evolution and clinical outcome appear to be similar [26].

JAK2 signaling is also constitutively activated by acquired mutations in *MPL* encoding the thrombopoietin receptor, and calreticulin (*CALR*), a calcium-binding chaperone of the endoplasmic reticulum. Given their redundant effect, *MPL* and *CALR* mutations rarely co-occur with *JAK2* mutations. For *MPL*, missense mutations of codon 515 (W515K or W515L) in exon 10 are most common and result in constitutive receptor activation inducing overactive JAK2 signaling [29]. *MPL* mutations are found in 3–5% of patients with ET and PMF, but not PV [30]. Mutations of *CALR* affect exon 9 and can mainly be assigned to two major categories [31,32]. In type 1, a deletion of 52 base pairs occurs, while type 2 is characterized by a five base pair insertion. Both types of *CALR* mutations induce a frameshift of one base pair, which results in the loss of the C-terminal KDEL sequence acting as an ER retention signal. Mutant CALR is secreted by the ER, associates with the thrombopoietin receptor, and can be detected at the cell surface, a finding that may prove useful for therapeutic approaches [33,34]. *CALR* mutations occur in 20–35% of patients with ET or PMF and are associated with more pronounced thrombocytosis, younger age, fewer thromboembolic events, and an overall more favorable prognosis compared to *JAK2* V617F mutant patients [35]. It is notable that even so-called “triple-negative” MPN patients who lack a driver mutation in *JAK2*, *CALR*, or *MPL* show activated JAK2 signaling, highlighting the significance of the JAK2 signaling network for MPN pathogenesis [36]. This finding also provides a rational basis for JAK2 inhibitor treatment in triple-negative MPN patients.

Apart from its profound impact on signaling, mutant JAK2 has also been shown to enter the nucleus and elicit epigenetic effects [37]. *JAK2* V617F phosphorylates histone H3, which prevents binding of heterochromatin protein 1α and results in altered gene expression [38]. Phosphorylation of protein arginine methyltransferase 5 (PRMT5) has analogous effects via decreased methylation of histone H2A and H4 [39]. PRMT5 inhibitors, either as a single agent or in combination with JAK1/2 inhibitors, reduce myeloproliferation in cell lines and mouse models [40].

In addition to constitutive activation of JAK2 signaling, persistent inflammation is increasingly recognized as an important driver of MPN development and progression [41]. *JAK2* V617F-mutated hematopoietic stem cells show increased expression of inflammatory cytokines such as TNFα, IFNα, and TGFβ. A relationship between chronic inflammation and clonal development was corroborated by the finding that TNFα promotes clonal expansion of *JAK2* V617F-mutated cells, while the absence of TNFα in *JAK2* V617F-transduced bone marrow cells was able to abolish the MPN phenotype in murine models [42]. Additionally, mutant *JAK2* has been reported to induce elevated levels of reactive oxygen species (ROS) by downregulating catalase expression, which leads to elevated levels of reactive H_2_O_2_ [43]. The persistent inflammatory milieu in MPN has been shown to promote bone marrow fibrosis and constitutional symptoms, correlating with an unfavorable prognosis [44]. Thus, liquid biopsy-based biomarkers of inflammatory activity may prove useful for diagnostic and prognostic purposes in the future, such as in distinguishing MPN subtypes and assessing the risk of thrombotic events [45].

## 4. Clinical Presentation of MPN

Philadelphia chromosome-negative myeloproliferative neoplasms (MPN) are clonal hematopoietic stem cell disorders and, based on activated JAK2 signaling, present with an excessive output of mature blood cells of one or more myeloid lineages [46]. When first described by William Dameshek in 1951, a hitherto unrevealed stimulus leading to proliferation of bone marrow cells was suggested to drive the MPN including PV, ET, and PMF [47]. While PV is characterized by erythrocytosis and potential thrombocytosis and neutrophilia, the hallmark of ET is isolated thrombocytosis. Primary myelofibrosis is characterized by enhanced, atypical megakaryopoiesis and cytoses in the pre-fibrotic phase and progressive bone marrow fibrosis and cytopenia in more advanced phases. Extramedullary hematopoiesis leading to hepatosplenomegaly is typical for all MPN and most pronounced in PMF, including the appearance of myeloid and erythroid precursors in the peripheral blood (leuko-erythroblastosis). Beyond the characteristic alterations of peripheral blood counts and bone marrow architecture, the *JAK2*, *CALR,* and *MPL* driver mutations have become biomarkers for the diagnosis of MPN and have been incorporated into the WHO classification of hematopoietic malignancies [46]. Specifically, the *JAK2* V617F mutation is instrumental in differentiating polycythemia vera from secondary erythrocytosis. It is still incompletely understood how PV, ET, or MF phenotypes may arise upon presence of the same *JAK2* V617F mutation. Several factors have been implicated, such as *JAK2* V617F mutant allele burden, which is usually lower in ET and higher in PV and MF, as well as loss of heterozygosity by mitotic recombination at chromosome 9p, leading to homozygosity for *JAK2* V617F. Furthermore, the acquisition order of the *JAK2* V617F and additional non-driver mutations, such as in the *TET2* gene, was shown to play a role in determining clinical MPN phenotypes [3,48,49].

The most frequent complications of MPN are thrombo-hemorrhagic events. MPN patients are at risk for both arterial and venous thromboses, affecting approximately 40% of patients with PV during the course of disease. Particularly, there is an increased susceptibility for thromboses in the hepato-lienal and portal venous systems, and splanchnic thrombosis are typically seen [50]. It has become clear that the prothrombotic condition in MPN relates to multiple factors including erythrocytosis, leukocytosis, activation of platelets, but also alterations in plasmatic coagulation and NET formation. In addition, inflammatory stimuli are known to induce endothelial dysfunction with pro-adhesive surface properties, which facilitates leukocyte and platelet binding and activation [51]. *JAK2* V617F expression in endothelial cells appears to promote such functional changes and an overall inflammatory state of the vasculature [52]. Interestingly, *JAK2* V617F is associated with a higher thrombotic risk than *CALR* mutations, and influences of the mutant cell fraction have also been shown [53]. Bleeding events are an additional concern and can relate to acquired von Willebrand syndrome due to preferential consumption of large von Willebrand factor (VWF) multimers in settings of excessive thrombocytosis with counts > 1000–1500 G/L.

While both PV and ET can progress to secondary myelofibrosis referred to as Post-PV/Post-ET MF, the risk of transformation to secondary acute myeloid leukemia (AML) is highest in PMF (10-year risk 10–20%), followed by PV (2–4%) and ET (approximately 1%) [54]. Outcomes are generally poor, reflecting the prevalent unfavorable cytogenetic and molecular features, which render the leukemic clones less sensitive to standard chemotherapeutic agents. Leukemic transformation can arise from the *JAK2* V617F mutant hematopoietic stem/progenitor compartment, but may also present as *JAK2* V617F-negative AML. Leukemic blasts preferentially show mutations in one of the two isoforms of isocitrate dehydrogenase (*IDH1*, *IDH2*), the tumor suppressor gene *TP53*, or serine and arginine-rich splicing factor 2 (*SRSF2*) (Table 1) [55,56,57]. Risk of leukemic transformation is associated with the overall number of acquired mutations, and the vast majority of these are already detectable at MPN diagnosis [58].

## 5. Clinical Benefit of JAK Inhibitor Therapy in MPN

Constitutive activation of JAK2 signaling in *JAK2*, *CALR,* and *MPL* mutant settings as well as in triple-negative MPN has provided a rational basis to develop JAK2 inhibition as a therapeutic approach in MPN [34]. Current JAK2 inhibitors that are approved (ruxolitinib, fedratinib) or in clinical development (momelotinib, pacritinib, etc.) engage the ATP-binding site of JAK2 in the active conformation, thereby interfering with JAK2 catalytic activity, and are referred to as type 1 inhibitors [59].

Ruxolitinib, which acts as a JAK1/2 inhibitor, represents a clinical standard of care for patients with intermediate or high-risk MF with symptomatic splenomegaly or constitutional symptoms. As demonstrated by the COMFORT trials, ruxolitinib is able to improve splenomegaly and symptom burden along with reduced cytoses and proinflammatory cytokine levels. A survival benefit was observed after one year of treatment [60,61]. Based on the RESPONSE study, ruxolitinib has also been approved for patients with PV resistance or intolerant to hydroxyurea [62] and is effectively reducing hematocrit, splenomegaly, and symptom burden. Ruxolitinib therapy also resulted in a lower frequency of thromboembolic events relating to reduced hematocrit and inflammation [63]. In ET, ruxolitinib has not shown additional clinical value so far [64].

Fedratinib, a second JAKinib with JAK2/FLT3 inhibitory activity, has recently been approved after clinical development was delayed by a clinical hold based on suspected cases of Wernicke’s encephalopathy. Analogous to ruxolitinib, fedratinib is able to reduce symptom burden and splenomegaly in JAK inhibitor-naïve patients as well as in patients who lose response or are intolerant to ruxolitinib, thus providing a second line JAK2 inhibitor treatment after ruxolitinib failure [65]. Monitoring of thiamine levels and/or supplementation is recommended and additional safety data from the phase 3b FREEDOM study are currently accumulating.

Several JAK2 inhibitors are in clinical development, which could prove beneficial in patients with anemia and/or thrombocytopenia. Pacritinib is a JAK2/FLT3 inhibitor with milder myelosuppressive properties and favorable results in patients with thrombocytopenia [66]. In addition, momelotinib represents a JAK1/2 inhibitor with additional impact on the activin A type 1 receptor (ACVR1), which favorably affects iron homeostasis and translates into reduced transfusion requirements [67,68]. Pacritinib and momelotinib are currently being evaluated in clinical trials and will hopefully soon extend our therapeutic options for cytopenic MPN patients.

Beyond MPN, the JAK1/2 inhibitor ruxolitinib has also shown activity in the therapy of glucocorticosteroid-refractory acute or chronic graft-versus-host disease [69,70]. Furthermore, immunosuppressive properties of JAK1/2 inhibitors are increasingly used to treat various autoimmune disorders, particularly rheumatoid arthritis.

## 6. Limitations of JAK Inhibitor Therapy in MPN

Current JAK2 inhibitor therapies in MPN show limited disease-modifying potential as an important limitation. Reductions in mutant allele burden are modest, and it has been demonstrated that clonal evolution is progressing during treatment with, e.g., ruxolitinib [71]. Continued therapy may lead to a certain decrease in clone size, but partial molecular remission, defined as a 50% reduction in *JAK2* V617F allele burden in patients with at least 20% mutant allelic burden at baseline, is achieved in <10% of patients. Therefore, the development of more effective therapeutic approaches with disease-modifying potential is imperative.

Anemia, thrombocytopenia, and to a lesser extent immunosuppression represent frequent on-target toxicities of JAK inhibitor treatment and may require dose modifications [61,72]. While anemia is most pronounced at treatment initiation, it may stabilize over time.

JAK1 inhibitory activity of ruxolitinib has been related to reduced immune surveillance and the association with higher incidences of opportunistic infections and particularly reactivation of Herpes zoster, but also reactivation of tuberculosis, cryptococcal meningoencephalitis, *Pneumocystis jirovecii* pneumonia, hepatitis B, toxoplasmosis, or cytomegalovirus retinitis have been reported underscoring the importance of increased vigilance [73,74,75,76,77]. In addition, it has been speculated that MPN patients treated with ruxolitinib could be at higher risk for secondary cancers, and a trend for increased occurrence of non-melanoma skin cancer has been observed in long-term studies [78]. Furthermore, aggressive B-cell lymphomas have been reported, particularly in patients with MPN and preexisting B-cell clones, but the data are conflicting [79,80].

Loss of response to JAK inhibitor therapy can occur over time and is seen in approximately 50% of MF patients who initially responded to therapy after 5 years of treatment [81]. Second-site mutations inducing resistance by interference with drug binding have been described in MPN cell lines but have not been reported in MPN patients. Thus, resistance to JAK inhibitors appears to relate to adaptive processes, which is highlighted by the observation that patients can regain response upon re-exposure after a drug holiday [82]. While reactivation of JAK2 signaling via JAK family heterodimer formation has been described [83], additional mechanisms of acquired resistance to JAK2 inhibition have also been reported [84].

## 7. Potential Future Avenues for JAK2 Inhibitor Development and Alternative Therapeutic Approaches

While current JAK2 inhibitors in clinical development all act via ATP competitive binding to the active conformation of the JAK2 kinase domain (type 1 inhibition), perspectives for the future development of enhanced JAK2 inhibitors include several alternative approaches. Selective inhibition of *JAK2* V617F represents an ultimate goal and would hopefully facilitate efforts to reduce MPN clone size. Type 2 JAK2 inhibition, which interferes with JAK2 in the inactive conformation, has shown promising effects on mutant allele burden in preclinical studies [85,86]. A different concept is the design of JAK2 pseudokinase inhibitors displacing ATP from the JAK2 JH2 domain. While minor effects on wild-type JAK2 have been detected, JH2 ATP binding appears to be critical for aberrant constitutive activity in *JAK2* V617F [87]. Promising results are accumulating for combining JAK2 inhibition with the targeting of additional factors involved in MPN pathogenesis. Such dual approaches, which combine JAK2 inhibitors with Bcl-2/Bcl-xL inhibition, bromodomain inhibition, interferon-alpha, PI3K pathway, MAPK/ERK pathway inhibition, or others, are in clinical evaluation and highlight the significance as well as the promise of therapeutic developments in MPN [88,89,90,91,92,93].

## 8. *JAK2* V617F in Clonal Hematopoiesis of Indeterminate Potential

Beyond MPN, the *JAK2* V617F mutation has recently been identified in clonal hematopoiesis of indeterminate potential (CHIP), a pre-malignant state that relates to the occurrence of a hematopoietic clone with somatic mutations known to be associated with myeloid neoplasms, but in a healthy individual with normal blood counts. The emergence of CHIP originates from a hematopoietic stem cell and is an age-dependent process, affecting approximately 10% of individuals over 70 years of age. Prevalence significantly depends on the applied method and its sensitivity to detect variant allele frequencies and may therefore be higher [94].

Along with mutations in *DNMT3A*, *TET2*, *ASXL1*, *SRSF2*, and *SF3B1, JAK2* V617F is among the most frequent mutations in CHIP [95]. Individuals carrying CHIP exhibit increased all-cause mortality, which is primarily attributable to a higher incidence of cardiovascular events rather than the elevated risk of developing hematopoietic neoplasms. The risk of developing coronary heart disease or ischemic stroke within 10 years was reported as roughly twofold in carriers of CHIP. In the presence of CHIP with an activating *JAK2* mutation, the risk of coronary heart disease was even further increased and reported to be approximately 12-fold [94]. The presence of CHIP is an independent cardiovascular risk factor and comparable to established risk factors, such as dyslipidemia, arterial hypertension, smoking, and diabetes mellitus. Likewise, venous thromboembolic events represent both initial manifestations of an underlying MPN but may also be associated with a *JAK2* V617F mutation without the clinical phenotype of a concomitant myeloid neoplasm. In a series of patients with normal blood counts, absence of panmyelosis, and apparently idiopathic hepatic venous thrombosis (Budd–Chiari syndrome), 50% harbored a *JAK2* V617F mutation. Moreover, 25% of all patients developed an MPN during follow-up, with ET diagnosed in approximately 75% [96]. While insight into the biology and clinical relevance of *JAK2* mutant CHIP is accumulating, questions regarding management and potential therapy are currently not clarified and warrant specific investigation in future studies.

## 9. Conclusions

Since the discovery of the *JAK2* V617F mutation, insight into the molecular pathogenesis of MPN has impressively increased and has provided a rational basis for the development of targeted therapy approaches. Novel JAK2 inhibitors and combination therapies are expected to become part of clinical practice. JAK2 remains central to disease understanding and a target in MPN.

## Figures and Tables

**Table 1:** Somatic mutations in myeloproliferative neoplasms (MPN). Mutation frequencies are indicated for polycythemia vera (PV), essential thrombocythemia (ET), and primary myelofibrosis (PMF) [27,28]. *JAK2*, *CALR,* and *MPL* mutations are considered as driver mutations, while so-called high molecular risk (HMR) mutations confer adverse prognosis in myelofibrosis. BP-MPN blast phase MPN.

Gene/Mutation	Chromosome	Mutational Frequency (%)		
		PV	ET	PMF
Driver mutations in MPN				
*JAK2* V617F (exon 14)	9p24	95	50–60	50–60
*JAK2* exon 12 mutations	9p24	2–3	-	-
*CALR*	19p13.2	<1	20–30	20–35
*MPL*	1p34	<1	1–5	5–9
High molecular risk (HMR) mutations in MF				
*ASXL1*	20q11.1			25–35
*EZH2*	7q36.1			1–10
*SRSF2*	17q25.1			10–18 (enriched in BP-MPN)
*IDH1/IDH2*	2q33.3/15q26.1			1–6 (enriched in BP-MPN)
Other mutations enriched in blast phase MPN				
*TP53*	17p13.1			1–5 (enriched in BP-MPN)

## Data Availability

Not applicable.

## Supplementary Materials

The following are available online at https://www.mdpi.com/article/10.3390/ph15020160/s1.
