# Supplementary material for: JAK2 in Myeloproliferative Neoplasms: Still a Protagonist
Source: Pharmaceuticals (Basel). 2022 Jan 28;15(2):160. doi: 10.3390/ph15020160 (PMC8874480; doi:10.3390/ph15020160)
Supplement: Supplementary file 1 [file pharmaceuticals-15-00160-s001.zip › pharmaceuticals-1555246-supplementary.pdf]

pharmaceuticals

Supplemental information to "JAK2 in Myeloproliferative Neoplasms: Still a Protagonist"

Michael Stephan Bader [1] and Sara Christina Meyer [1,2,*]

[1]Division of Hematology, University Hospital Basel, and [2]Department of Biomedicine, University Hospital Basel and University of Basel, Switzerland, * corresponce to: sara.meyer@unibas.ch

Supplemental methods

To retrieve the relevant literature to the scope of this review article, we performed a literature search in PubMed (including search terms JAK2, JAK-STAT signaling, myeloproliferative neoplasms, JAK2 inhibition, JAK2 inhibitor, JAK inhibitor resistance, and JAK inhibitor failure). We considered articles published in English preferentially in the last five years and extended the inclusion period to ten years for important original reports. Key papers in the field published in top journals were also referenced when published before 2011 to provide the direct information on landmark papers to the readers.
